# Vitamin K deficiency: the linking pin between COPD and cardiovascular diseases?

**DOI:** 10.1186/s12931-017-0673-z

**Published:** 2017-11-13

**Authors:** Ianthe Piscaer, Emiel F. M. Wouters, Cees Vermeer, Wim Janssens, Frits M. E. Franssen, Rob Janssen

**Affiliations:** 1 Department of Respiratory Medicine, Maastricht University Medical Center+, Maastricht, The Netherlands; 2 CIRO, Center of Expertise for Chronic Organ Failure, Horn, The Netherlands; 3 R&D Group VitaK, Maastricht University, Maastricht, The Netherlands; 4 Department of Chronic Diseases, Metabolism and Ageing, Laboratory of Respiratory Disease, University of Leuven, Leuven, Belgium; 5 Department of Pulmonary Medicine, Canisius-Wilhelmina Hospital, Nijmegen, The Netherlands

**Keywords:** COPD, Cardiovascular diseases, Desmosine, Elastin, Matrix Gla protein, Vascular calcification, Vitamin K, Vitamin K antagonists

## Abstract

Cardiovascular diseases are prevalent in patients with chronic obstructive pulmonary disease (COPD). Their coexistence implies that many COPD patients require anticoagulation therapy. Although more and more replaced by direct oral anticoagulants, vitamin K antagonists (VKAs) are still widely used. VKAs induce profound deficiency of vitamin K, a key activator in the coagulation pathway. It is recognized however that vitamin K is also an essential cofactor in the activation of other extrahepatic proteins, such as matrix Gla protein (MGP), a potent inhibitor of arterial calcification. No or insufficient MGP activation by the use of VKAs is associated with a rapid progression of vascular calcification, which may enhance the risk for overt cardiovascular disease. Vitamin K consumption, on the other hand, seems to have a protective effect on the mineralization of arteries. Furthermore, vascular calcification mutually relates to elastin degradation, which is accelerated in patients with COPD associating with impaired survival. In this commentary, we hypothesize that vitamin K is a critical determinant to the rate of elastin degradation. We speculate on the potential link between poor vitamin K status and crucial mechanisms of COPD pathogenesis and raise concerns about the use of VKAs in patients with this disease. Future intervention studies are needed to explore if vitamin K supplementation is able to reduce elastin degradation and vascular calcification in COPD patients.

## Background

Cardiovascular diseases are more prevalent in patients with chronic obstructive pulmonary disease (COPD) compared to age- and smoking-matched controls with no lung disease [1]. Vascular calcification is a major risk factor for cardiovascular morbidity and mortality. COPD patients have on average more extensive coronary artery calcification (CAC) than controls [2]. Furthermore, the burden of emphysema is related to the thoracic aortic calcification score [3]. The frequency of cardiac arrhythmias is also high in patients with COPD [1], and an inverse association has been identified between forced expiratory volume in one second and incident atrial fibrillation [4]. Atrial fibrillation and pulmonary embolism may be both cause and consequence of acute COPD exacerbations, and often necessitate prolonged anticoagulation therapy [5, 6].

Although the use of direct oral anticoagulants (DOACs) is rising, vitamin K antagonists (VKAs) are still widely used as anticoagulant drugs. VKAs inhibit vitamin K recycling thereby inducing functional vitamin K deficiency [7, 8]. Vitamin K is generally known as an activator of coagulation proteins in the liver and therefore often incorrectly regarded as a mono-functional cofactor [9]. It is much less acknowledged that vitamin K is also essential in the activation of extrahepatic key-proteins [9]. Matrix Gla protein (MGP) is vitamin K-dependent and a potent inhibitor of soft tissue calcification [10]. Furthermore, evidence suggests a potential role for MGP in the protection of extracellular matrix proteins from enzymatic degradation [11]. MGP knock-out mice die within two months after birth due to vascular calcifications leading to large blood vessel rupture, illustrating the importance of MGP [10]. Although research has mainly focused on its protective effects against arterial pathologies [12], MGP is also extensively expressed in the lungs [13].

### Vitamin K status

Vitamin K cannot be produced endogenously and is exclusively obtained exogenously. Different forms of vitamin K can be discerned, including naturally occurring vitamins K1 and K2 [14]. Vitamin K2 usually comprises not more than about one-tenth of total vitamin K consumption, but it holds a much larger share in the activation of vitamin K-dependent proteins as vitamin K2 has higher bioavailability and longer half-life time than K1 [14]. Although there is no absolute tissue specificity, vitamin K1 is preferentially used in the liver to activate coagulation factors, whereas vitamin K2 has a more prominent role in the activation of extrahepatic vitamin K-dependent proteins, such as MGP [15].

Vitamin K1 levels can be reliably measured in the circulation and reflect the intake of vitamin K1 [16]. Vitamin K2, however, usually cannot be detected in the blood stream unless taken as supplements [16]. To date, there is no gold standard for assessing total vitamin K status, although measuring inactive levels of vitamin K-dependent proteins in the circulation seems to be the most appropriate method [16]. Desphospho-uncarboxylated (dp-uc; i.e. inactive) MGP levels are often used as a surrogate marker for vitamin K status. Dp-ucMGP levels are inversely correlated with vitamin K status, which means that subjects with high dp-ucMGP levels have low vitamin K status and vice versa [16].

**Fig. 1:**
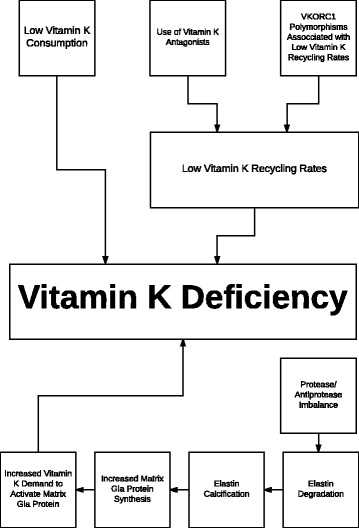
Proposed mechanisms that could be responsible for vitamin K deficiency. Low vitamin K consumption and use of vitamin K antagonists induce vitamin K deficiency. It is likely that polymorphisms in vitamin K epoxide reductase complex subunit 1 (VKORC1) gene associated with low vitamin K recycling rates predispose to vitamin K deficiency. Accelerated elastin degradation, due to a protease/antiprotease imbalance, leads to elastin calcification and subsequently to an increased synthesis of matrix Gla protein, which needs to be activated by vitamin K. This increased vitamin K demand might also cause a vitamin K deficit

There are several potential reasons why vitamin K status might be impaired (Fig. 1). Obviously, it can be the result of low vitamin K consumption. Cheese is an important source of vitamin K2 in many countries. In relation to COPD, it is interesting that cheese consumption was shown to be associated with better lung function and less emphysema in a large observational study [17].

**Fig. 2:**
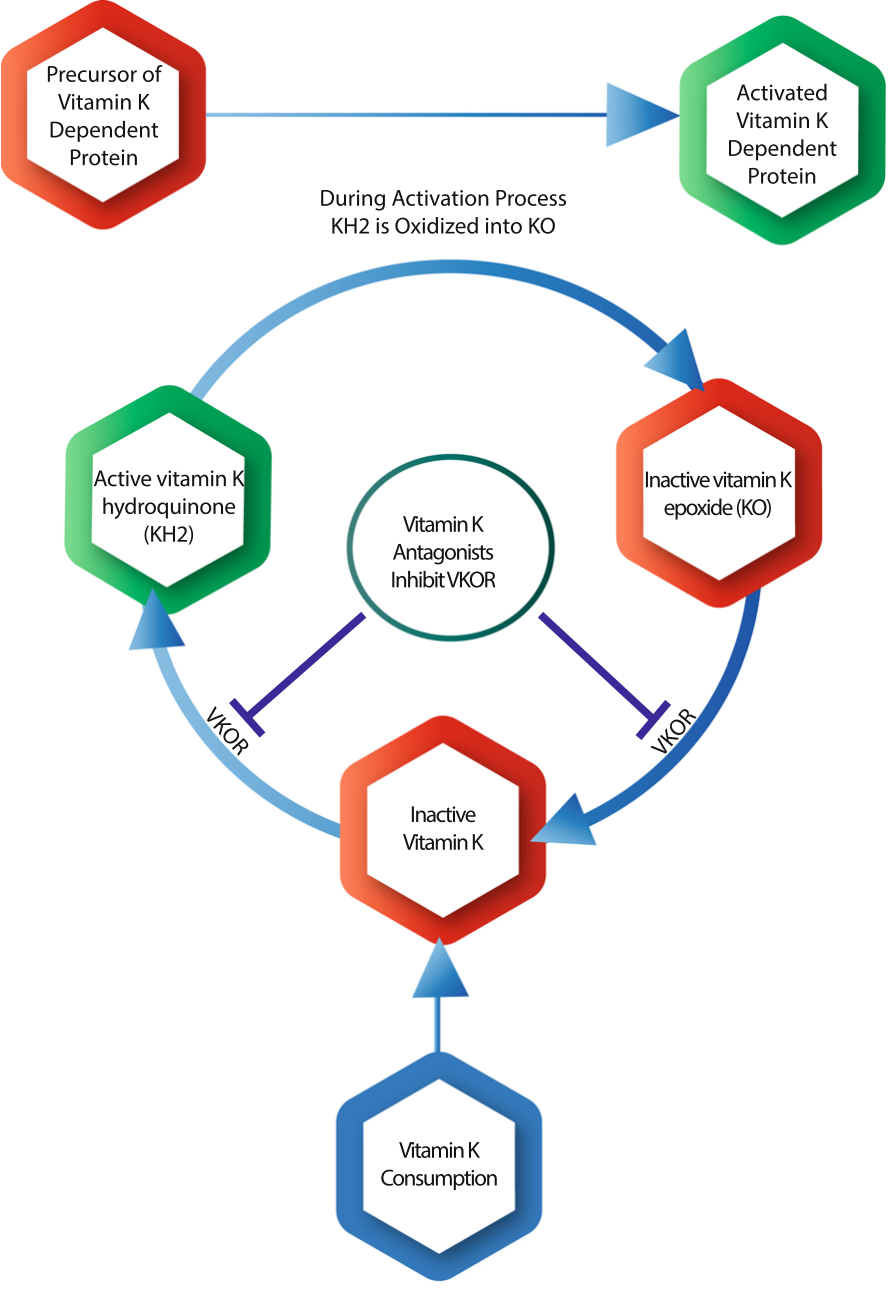
Vitamin K cycle. Food-derived vitamin K first needs to be converted into the active metabolite vitamin K hydroquinone (KH_2_). During the activation process of vitamin K-dependent proteins, the cofactor KH_2_ is converted into vitamin K epoxide (KO). Subsequently, inactive KO has to be reduced, first into vitamin K and then into KH_2_. These two reduction steps are executed by the enzyme vitamin K epoxide reductase (VKOR). VKAs are specific inhibitors of VKOR

Differences in vitamin K metabolism due to the use of VKAs or genetic variation are other causes of a lower vitamin K status. The human body uses vitamin K very economically given that it is reused about 2000 fold via the so-called vitamin K cycle (Fig. 2). The two reduction steps of this cycle are executed by the enzyme vitamin K epoxide reductase (VKOR). VKAs are specific inhibitors of VKOR [7], which is the explanation for the poor vitamin K status found in subjects using these anticoagulant drugs [8]. The VKOR activity is also influenced by single nucleotide polymorphisms (SNPs) in the VKOR complex subunit 1 (VKORC1) gene, which are in linkage disequilibrium with each other [7]. VKORC1 SNPs that are associated with low vitamin K recycling rates may be overrepresented in subjects with low vitamin K status. The T-allele of the VKORC1 1173C > T SNP associates with poor vitamin K recycling and a significantly higher risk of aortic calcification [18]. We could further speculate that the VKORC1 C1173T SNP might also influence the susceptibility to COPD or specific phenotypes such as emphysema. Genetic association studies are needed to assess whether this hypothesis holds true

Finally, it is possible that subjects with enhanced rates of elastin degradation have higher vitamin K expenditure given that elastin degradation stimulates elastin calcification [19]. A rising calcium content in elastin fibers stimulates MGP synthesis in an attempt to prevent further calcium precipitation within the elastin fibers [20]. However, MGP first needs to be activated by vitamin K. This increased vitamin K demand might induce a vitamin K deficit.

### Elastin calcification and degradation

Vascular calcification usually starts in the elastin fibers of the arterial medial wall [19]. Elastin is a unique protein that provides elasticity, resilience and deformability to dynamic tissues, such as arteries and lungs [21]. It is mainly produced in utero and early childhood after which elastin synthesis is suppressed at a posttranscriptional level [21]. During the aging process, the elastic properties of elastin fibers can be compromised by both calcification and degradation [21]. Elastin has high affinity for calcium, and as a result its calcium content increases during aging [22]. Arterial calcifications can be induced in rats via the administration of VKAs to induce vitamin K deficiency and thereby preventing vitamin K-dependent MGP activation [11]. A similar mechanism has also been demonstrated in humans in whom the use of VKAs is associated with more vascular calcifications [23, 24]. This is of course highly undesirable in calcification-prone COPD patients.

Elastin degradation is enhanced in patients with COPD due to an imbalance between the protective effects of antiproteases and the destructive properties of proteases. In COPD patients, elastinolysis is not only accelerated in lungs but also in other elastin-rich tissues [25]. The severity of emphysema is correlated with arterial stiffness and skin wrinkling, indicating that a process of “systemic elastin degradation” may occur in COPD [25]. Desmosine and isodesmosine (DES) are two amino acids that are only present in crosslinked elastin fibers, and plasma DES levels therefore reflect the rate of elastin degradation. The COPD Biomarker Qualification Consortium regards DES as biomarkers with great potential [26]. In a uniform cohort of COPD patients with alpha-1 antitrypsin (AAT) deficiency, plasma DES was related to emphysema progression [27]. Plasma DES levels were not associated with emphysema in a cohort of patients with a variety of COPD endotypes and phenotypes [28]. However, they were correlated with arterial stiffness and cardiovascular comorbidity in these heterogeneous COPD patients [28], which probably indicates that the vascular compartment is the main contributor of elastin degradation products in the blood stream of most AAT sufficient COPD patients. Remarkably, plasma DES levels were also associated with the CAC score, illustrating the close relationship between elastinolysis and vascular calcification [28].

Elastin calcification and degradation are two pathogenic mechanisms that stimulate each other [19]. Administration of calcium chloride (CaCl_2_) in rat aortas induced both elastin calcification and degradation [19]. Inhibiting elastin degradation, prior to CaCl_2_ administration, also reduced elastin calcification [19]. The calcification promoting actions of elastases are probably based on the higher affinity of degraded elastin for calcium than intact elastin probably due to increased polarity of the former [22]. Elastin calcification, on the other hand, induces an upregulation of matrix metalloproteinase (MMP) gene expression leading to an acceleration of elastin degradation [29]. In an animal model, it has been demonstrated that VKAs not only induced elastin mineralization but also promoted elastin degradation [11]. MMP-9 activity even preceded macroscopic elastin calcification [11]. The interrelationships between elastin calcification, elastin degradation and the actions of MGP could potentially be a pathomechanistic explanation for the observed link between COPD and cardiovascular diseases.

### Osteoporosis and vascular calcification

Remarkably, demineralization of bone tissue and mineralization of arteries often coexist in individual patients with COPD [30, 31]. The mechanism behind this apparently paradoxical link has yet to be established. We speculate that insufficient activation of the vitamin K-dependent proteins osteocalcin (OCN) and MGP might be involved. Whereas activated OCN is regarded as a regulator of mineralization in bone tissue, MGP protects extra-osseous tissues from calcification. This concept is supported by a randomized-controlled trial in postmenopausal women demonstrating that vitamin K2 supplementation ameliorated both bone loss and arterial stiffening [32, 33]. Future studies in well-characterized cohorts have to reveal whether vitamin K deficiency is indeed the linking pin between osteoporosis and vascular calcifications in COPD.

### Vitamin K supplementation

Preliminary data of our group suggest that vitamin K status is reduced in patients with COPD compared to controls [34]. We also found an inverse association between vitamin K status and plasma DES levels; i.e. lower vitamin K status related to accelerated elastin degradation [34].

The rate of elastin degradation seems to be a strong independent predictor of mortality in COPD [28]. Decelerating elastin degradation might therefore be an attractive therapeutic target in COPD. A recent trial, in which AAT augmentation therapy reduced plasma DES levels in COPD patients with AAT deficiency, serves as a convincing proof-of-principle for this intriguing concept [27]. Since vitamin K supplementation can be hypothesized as an alternative therapeutic strategy, an intervention trial should be conducted to assess whether vitamin K supplementation does indeed decelerate elastin degradation in patients with COPD.

**Fig. 3:**
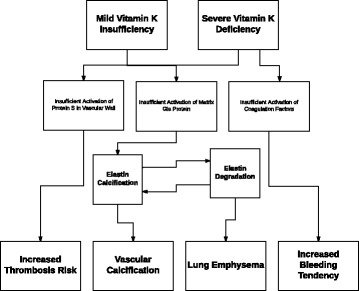
Triage theory. The triage theory implies that in case of mild vitamin K deficiency the coagulation factors are still activated by vitamin K, however, the anticoagulation protein S in the vascular wall and matrix Gla protein are insufficiently activated. This will lead to both increased thrombosis risk and elastin calcification. Elastin calcification causes elastin degradation and vice versa. Elastin degradation in the lungs leads to lung emphysema. Vascular calcification begins at the elastin fibers in the vascular walls. Only in case of severe vitamin K deficiency, coagulation factors are also insufficiently activated leading to increased bleeding tendency

Vitamin K has an excellent safety profile, and no toxicity has been observed even with very high-doses [35]. Vitamin K is obligatory to maturate clotting factors in the liver, however, it is also necessary for the activation of anticlotting factors (i.e. protein C and S). Whereas protein C is solely produced in the liver, about 50% of protein S is synthesized hepatically and the other half in the vascular wall [36]. Protein S production in the vascular system seems to be of key importance in local thrombosis prevention [36]. The triage theory posits that in case of scarcity, nature will provide nutrients first to places in the body where shortage leads to an immediate treat to short-term survival at the expense of places where shortage only has long-term consequences (Fig. 3) [37]. Regarding vitamin K deficiency, increased bleeding tendency is the biggest short-term threat for survival, and the limited supply of vitamin K will therefore be preferentially used for the synthesis of clotting factors with the sacrifice of protein S maturation in the vascular wall [37]. Therefore, counterintuitively, vitamin K supplementation does not increase the risk of thromboembolism and might even decrease it by fully activating the anti-thrombosis activity [37]. The triage theory also implies that MGP activation in VKA-users is more severely compromised than the activation of coagulation factors [38], which might have deleterious effects on vascular calcifications, elastin degradation and survival in patients with COPD.

### Vitamin K1 and K2 in cardiovascular diseases

Whether vitamin K2 alone or both vitamin K1 and K2 have beneficial effects on vascular calcification remains partially elusive. Data from observational studies suggest that vitamin K2 rather than vitamin K1 has protective properties against cardiovascular morbidity and mortality [39, 40]. Participants in the non-interventional Rotterdam study were categorized based on the estimated consumption of vitamin K1 and K2 [39]. Subjects from the upper vitamin K2 intake tertile had 0.57 lower relative risk of cardiovascular mortality than those from the lowest one [39]. The presence of severe aortic calcification in the highest tertile for vitamin K2 intake was also less than 50% compared to that in the lowest tertile [39]. Vitamin K1 was not related to either mortality or aortic calcification in the Rotterdam study [39]. The suggestion of a protective effect of vitamin K2 consumption and an indifferent effect of vitamin K1 on vascular calcification was corroborated in the observational Prospect-EPIC study [40]. More than 16,000 women were followed for on average 8 years, and, unlike higher vitamin K1 intake, higher intake of vitamin K2 was associated with reduced incidence of coronary heart disease [40].

Intervention trials, on the other hand, suggest a favorable effect of both vitamin K1 and K2 on cardiovascular pathology. A randomized-controlled trial assessed the effect of one year vitamin K1 supplementation on aortic valve calcification, which turned out to progress significantly slower in the active arm [41]. In another interventional trial, postmenopausal women were supplemented with vitamin K2 or placebo for three years [32]. Vitamin K2 improved arterial compliance, especially in those women with high stiffness at baseline [32]. Arterial stiffness is a strong independent predictor of cardiovascular risk [42], which is for a large part caused by the pathophysiological processes elastinolysis and elastocalcinosis [19]. COPD patients have increased stiffening of their arteries compared to controls [43]. Whether vitamin K supplementation has favorable effects on arterial stiffness in COPD is currently unknown. Future studies are needed to assess whether vitamin K could help to prevent cardiovascular diseases in patients with COPD.

In a rat model, equivalent doses of vitamin K1 and K2 were equally effective in reversing vascular calcifications [44]. Inactivation of MGP by the administration of VKAs led to rapid calcification of arteries in these animals [44]. Subsequently, both high-dose vitamin K1 and K2 supplementation reduced the calcium content in the rats’ arteries by some 50% [44].

It might be that the differences between the effects of vitamin K1 and K2 supplementation are dose-dependent. Given the lower vitamin K1 bioavailability and shorter half-life compared to that of K2, it is conceivable that higher doses of vitamin K1 are needed to achieve the same results on cardiovascular endpoints than with vitamin K2.

### Effect of vitamin K antagonists on elastin degradation

Since the rate of elastinolysis is related to mortality in COPD [28], we speculate that the stimulating effect of VKAs on elastin degradation might contribute to disease progression and mortality in patients with COPD. VKAs cause vitamin K deficiency leading to inadequate levels of active MGP to protect elastin fibers from calcification. MGP is virtually the only protein that is able to inhibit elastin calcification given other anti-calcifying proteins, such as fetuin, are too large to penetrate into the interior of the elastin fibers [12, 45]. Microscopic elastin calcification induces an increase of MMP synthesis leading to accelerated elastin degradation [29]. Preliminary data of our group suggest that VKAs have an accelerating effect on elastin degradation [46]. Plasma DES levels were higher in VKA-users compared to subjects not using these anticoagulant drugs [46]. However, additional studies are still required to unequivocally establish that VKAs enhance elastin breakdown.

### Alternative anticoagulant drugs in COPD

The use of VKAs has reduced in recent years following the introduction of DOACs. Contrary to VKAs, DOACs work directly on coagulation factors thrombin or factor Xa without interrupting the vitamin K cycle and the activation of MGP. It is therefore not to be expected that DOACs promote vascular calcification and/or elastin degradation. A variety of studies are currently registered on ClinicalTrials.gov comparing the effects of VKAs and DOACs on progression of vascular calcification [47]. Our group intends to conduct a trial assessing the differential effects of both anticoagulant drugs on elastolysis. These clinical studies are needed to unequivocally establish the effects of VKAs and DOACs on central mechanisms of COPD pathogenesis. With the current state of scientific evidence, however, it might already be argued that VKAs are better to be avoided in COPD patients given the established adverse effect of VKAs on arterial mineralization and the high prevalence of cardiovascular diseases in COPD [1, 23, 24].

### Future studies assessing the role of vitamin K in COPD pathogenesis

Additional animal and human studies are needed to fully unravel the role of vitamin K on the development and progression of COPD.

A variant of the smoking mouse model, previously applied to study the effects of vitamin D status on emphysema formation [48], could be used to assess the effects of high and low vitamin K status on lung destruction. Analogously to a rat model of vascular calcification, vitamin K deficiency is probably best induced in these experiments by combining high-dose VKAs to prevent vitamin K-dependent MGP activation with vitamin K1 to prevent hemorrhage [11].

Furthermore, large human cohort studies are needed to see if vitamin K deficiency is a significant factor for pulmonary disease progression in COPD. Intervention trials in patients with COPD are necessary to assess whether vitamin K administration may decelerate pulmonary elastin degradation and emphysema progression.

## Conclusions

Abundant circumstantial evidence, both from human observational studies and from animal intervention studies, points to a link between low vitamin K levels, elastin degradation and cardiovascular pathology in COPD patients. As VKAs are still widely used in this particular population concerns may rise on their long-term safety profile. Vitamin K intervention studies are warranted to reveal if vitamin K supplementation may play a role in the management of patients with COPD.

## Abbreviations

AAT: Alpha-1 antitrypsin
CAC: Coronary artery calcification
CaCl_2_: Calcium chloride
COPD: Chronic obstructive pulmonary disease
DES: Desmosine and isodesmosine
dp-uc: Desphospho-uncarboxylated (i.e. inactive)
KH_2_: Vitamin K hydroquinone
KO: Vitamin K epoxide
MGP: Matrix Gla protein
MMP: Matrix metalloproteinase
OCN: Osteocalcin
SNP: Single nucleotide polymorphism
VKA: Vitamin K antagonist
VKOR: Vitamin K epoxide reductase
VKORC1: VKOR complex subunit 1
